# As the virus evolves, so too must we: a drug developer’s perspective: We need a new paradigm in searching for next-generation countermeasures

**DOI:** 10.1186/s12985-022-01887-y

**Published:** 2022-10-10

**Authors:** Fang Flora Fang

**Affiliations:** Abimmune Biopharma, Inc., P.O. Box 8793, Rancho Santa Fe, CA 92037 USA

**Keywords:** COVID19, SARS-CoV-2, Host-directed therapeutics, HDTx, Host-directed antiviral, HAD, Immune evasion, Drug resistance, Pandemic countermeasures

## Abstract

The SARS-CoV-2 virus has been raging globally for over 2 years with no end in sight. It has become clear that this virus possesses enormous genetic plasticity, and it will not be eradicated. Under increasing selective pressure from population immunity, the evolution of SARS-CoV-2 has driven it towards greater infectivity, and evasion of humoral and cellular immunity. Omicron and its expanding army of subvariants and recombinants have impaired vaccine protection and made most antibody drugs obsolete. Antiviral drugs, though presently effective, may select for more resistant strains over time. It may be inevitable, then, that future SARS-CoV-2 variants will be immune to our current virus-directed countermeasures. Thus, to gain control over the virus, we need to adopt a new paradigm in searching for next-generation countermeasures and develop host-directed therapeutics (HDTx) and host-directed antivirals (HDA). Different from the virus-directed countermeasures, HDTx and HDA may offer variant agnostic treatment to reduce the risk and severity of infections. In addition, they may exert more uniform effects against the genetically diverse SARS-CoV-2 quasispecies, thereby diminishing the risk of selecting resistant variants. Some promising HDTx and HDA approaches are summarized here.

More than two years into the pandemic, it has become clear that SARS-CoV-2 will be a fact of life for the foreseeable future. Since early 2021, the world has witnessed more than 15 circulating SARS-CoV-2 variants, culminating with the ongoing Omicron outbreak. In spite of the pre-existing population immunity from vaccination and previous infections, Omicron has infected more people than all of the previous variants combined.

Overall, Omicron has increased the risk of SARS-CoV-2 reinfection by a factor of 16 [1]. Within 10 months, Omicron has grown into an ever enlarging collection of sublineages and recombinant variants. The Omicron outbreak is now a succession of overlapping waves beginning with BA.1, and followed by BA.1.1, BA.2, BA.2.12.1, BA.2.3, BA.2.9, BA.4, and BA.5. Since July 2022, BA.5 has replaced the BA.2 sublineages to become the dominant variant globally. Although BA.5 still represents 84% of the sequences when this article is being published, over 130 novel SARS-CoV-2 lineages and sublineages are now growing faster than BA.5 and are poised to replace it [2].

Being more transmissible than the Delta variant, Omicron BA.1 set a new record of transmissibility. This record has now been broken multiple times. An analysis of the 6.9 million SARS-CoV-2 genomes using Bayesian Viral Allele Selection (BVAS) has revealed that compared to Omicron BA.1, which has a relative growth rate of 1.6, BA.5 and BA.2.12.1 were growing over 4.7 times faster by having the relative growth rates of 7.8 and 7.9, respectively [3].

Coinciding with its increasing infectivity, SARS-CoV-2 has been gaining stronger binding to the ACE2 receptor as well. Although all Omicron sublineages have gained significantly higher affinity to ACE2 than the previous variants, the emerging BA.2.75 sublineage has profoundly surpassed them all by reaching 146 pM in K_D_ value [4]. A molecular docking simulation has revealed that compared to the ACE2 docking affinity of the ancestral Wuhan spike protein, the affinity of BA.5 spike has increased by 5 times, whereas the affinity of the BA.2.75 spike has increased by 21 times [5].

Clearly, SARS-CoV-2 has been evolving rapidly to adapt to the changing environment in the human population. The virus has confirmed our previous prediction that future SARS-CoV-2 variants may eventually breach the “protective ceiling” of the humoral immunity through dual escaping mechanisms: mutating the immune epitopes (epitope escape) and increasing the spike-ACE2 affinity (kinetic escape). As a result, all vaccines and antibodies could be rendered ineffective at preventing infections [6].

Underlying the tremendous capacity of SARS-CoV-2 to continuously evade immunity and enhance infectivity is the highly plastic nature of this virus. A stunning > 70% to 100% of the amino acid positions in the SARS-CoV-2 viral proteins carry mutations and can be replaced by an average of 2 to 4 different amino acids at each position [7]. In addition, SARS-CoV-2 is inherently prone to recombinations [8]. As SARS-CoV-2 becomes more genetically diversified, the impact of recombinations will be felt increasingly. Indeed, the first extraordinary increase in the emergence of SARS-CoV-2 recombinant lineages has happened during the Omicron wave [9].

Looking ahead, these factors, together with increasing selective pressure in the population, will cause the genomic diversification of SARS-CoV-2 to accelerate [7, 10]. The conventional virus-directed countermeasures, such as the variant-chasing vaccines, antivirals, or antibodies, will be chasing after an accelerating moving target.

Vaccines, especially mRNA vaccines, have made tremendous impacts on controlling the pandemic in 2020 and 2021. They are still the best way for reducing COVID19 hospitalization and death in a population. However, against the Omicron infections in 2022, immune protection from vaccination has become significantly impaired. Vaccine efficacy (VE) against breakthrough infections after the 3rd dose of the BNT162b2 mRNA vaccine was only 53.4% at its peak and further declined to 16.5% two months later [11].

Notably, it appears that the current mRNA vaccines may have hit a ‘ceiling of immunity’ after the 3rd dose. A study demonstrated that the 4th dose of BNT162b2 mRNA vaccine could at best temporarily boost VE against infection to 64% of the peak 3rd-dose level, which declined to 29% of the 3rd-dose level 7 weeks later, although VE against severe disease was maintained at > 73% of the 3rd-dose level for 10 weeks [12]. People on treatment for autoimmune diseases tend to respond poorly to vaccination and have a greater need for vaccine boosters. However, in this population, the 4^th^ dose of mRNA vaccine did not improve protection against Omicron and further, it caused flare-ups of autoimmune diseases in some patients [13]. For elderly people, low-quality immune response to vaccination seem to be caused by a T cell repertoire-intrinsic defect that may not be rescued by repeated vaccination [14].

Vaccination has also primed the population for immunity against the wild type (WT) SARS-CoV-2 virus which may compromise the Omicron specific booster vaccines. Consistently, for the 3-dose vaccinees, subsequent BA.1 infections mainly recalled neutralizing antibodies (NAbs) against the WT virus, although some new NAbs against BA.1 were also induced. These NAbs, however, are mostly evaded by BA.2, BA.4 and BA.5 [15].

On Aug 31, 2022, the FDA authorized bivalent booster vaccines that are updated against BA.4 and BA.5. These vaccines have not been subjected to clinical trials; thus their VE remains to be seen. In mice models, the BA.4/5 bivalent booster produced a modest increase (≤ 10 fold) of omicron-specific and cross-reactive NAbs that is similar to the BA.1 bivalent booster, and both of the bivalent boosters exhibited similar protection as the monovalent (Wuhan) booster against the BA.5 challenge in vivo [16].

Fortunately, current vaccines still confer significant protection against severe COVID19 as cellular immunity from vaccination wanes much slower. However, SARS-CoV-2 has been evolving towards evasion of cellular immunity too, albeit at a slower pace and mostly affecting individuals with certain genetic backgrounds [17, 18]. For example, T cell reactivity to an Omicron spike has reduced by > 50% in ~ 20% of individuals [19].

In late 2020 and early 2021, two antibody cocktail drugs (Etesevimab + Bamlanivimab & Imdevimab + Casirivimab) entered the market under EUA. In November 2021, Omicron BA.1 appeared and made these two drugs, as well as several antibody drug candidates still in development, obsolete. Only one drug, Sotrovimab, was still active. Three months later, as BA.2 replaced BA.1, Sotrovimab became obsolete [20]. Since then, two new antibody drugs, Cilgavimab/Tixagevimab and Bebtelovimab, have been granted EUA and they are effective against BA.2 in vitro [21]. However, BA.4 and BA.5 are significantly less sensitive to Cilgavimab/Tixagevimab compared to BA.2, which left Bebtelovimab the only drug equally effective against BA.2, BA.4 and BA.5 [22].

Recently, it was shown that by acquiring just one or two convergent mutations, both BA.2 and BA.5 can become profoundly resistant to the current antibody drugs, including Bebtelovimab [23]. Therefore, it may be a matter of time before Bebtelovimab loses its current status. Although broadly neutralizing antibodies that can inhibit all pre-circulated Omicron variants including BA.5 have been discovered and may offer some new hopes, it does not bode well that when subjected to in vitro selection the BA.5 spike protein became resistant to nearly all of the 40 broadly neutralizing antibodies after making just 2 to 3 changes in its sequence [24].

An inhibitor of the SARS-CoV-2 main protease (Mpro), Paxlovid, has now become the most common COVID19 treatment. Optimism over long-term utility of Paxlovid, however, is tempered by the fact that the “evolutionarily conserved” Mpro pocket targeted by Paxlovid and many other Mpro inhibitors in the current pipeline is in fact highly mutable. Deep mutational scanning has revealed that numerous mutations may cause resistance to the inhibitors with little effect on the Mpro function [25]. Furthermore, as the Mpro inhibitory candidates bind to the same or overlapping sequences in Mpro, they also share the same drug resistance vulnerability [26, 27]. Therefore, it is likely that once SARS-CoV-2 develops resistance to the first drug, Paxlovid, the current pipeline of Mpro inhibitors could become obsolete. In fact, among the circulating SARS-CoV-2 strains, there are already preexisting Paxlovid-resistant variants which are capable of spreading [28, 29].

Remdesivir (RDV) is a nucleoside analog inhibitor of the viral RNA dependent RNA polymerase (RdRp; nsp12 in SARS-CoV-2) that has been approved by the FDA for treatment of severe SARS-CoV-2 infections. At present, RDV resistant mutations are still rare among the 6 million published nsp12-RdRp consensus sequences. However, when subjected to RDV selection in cell cultures, SARS-CoV-2 readily developed RDV-resistant mutations, and the mutations are located either within the “evolutionarily conserved” active site of nsp12 or adjacent to the active site [30].

The situation of vaccines and antibodies is a testament to the uphill battle of keeping up with SARS-CoV-2 as it evolves to evade our current virus-directed countermeasures. As antiviral drugs are being increasingly used, it may be inevitable that SARS-CoV-2 would develop resistance to them as well. Thus, the rapid evolution of SARS-CoV-2 calls for a new hosting-directed paradigm and development of host-directed therapeutics (HDTx) and host-directed antivirals (HDA).

As an RNA virus, SARS-CoV-2 is prone to random mutations and recombinations. A SARS-CoV-2 population in an infection is a heterogenous mixture of genetically diverse quasispecies [31], each of which may have different susceptibility to a virus-directed countermeasure designed to target a single prototype sequence. As a result, a virus-directed countermeasure may exert inconsistent effects against different SARS-CoV-2 quasispecies, and the resistant quasispecies clones would gain growth advantage and be selected over time. By contrast, via modifying the host environment, HDTx and HDA drugs could level the playing field and exert more uniform effects against the SARS-CoV-2 quasispecies, thereby diminishing the risk of selecting resistant variants.

Via strengthening host defense against infections, or via modulating the pathogenic maladaptive inflammatory responses to infections, HDTx may reduce the risk of infection, alleviate the symptoms and organ damages, reduce hospitalization, morbidity, and mortality.

A number of HDTx immune modulating drugs have been used to treat the hyperactive inflammation associated with severe COVID19 in hospitalized patients, including corticosteroids (dexamethasone), IL-6 inhibitors (tocilizumab or sarilumab), and JAK inhibitors (baricitinib or tofacitinib). These drugs are recommended by the current COVID19 treatment guidelines for treatment of severe COVID19 in certain hospitalized patients [32]. In multiple clinical trials, Dexamethasone improved outcomes and reduced mortality in hospitalized patients with COVID19 [33]. In a randomized trial of hospitalized COVID19 patients, tocilizumab and sarilumab resulted similar ~ 20% reduction of 28-day mortality with ORs being 0·82 (0·71–0·95, p = 0·008) and 0·80 (0·61–1·04, p = 0·09) respectively [34].

In May 2022, baricitinib (Olumiant) was approved by the FDA for hospitalized COVID19 patients. In a phase 3 trial of hospitalized COVID19 patients, baricitinib monotherapy reduced death by Day 28 from 13.3% for the placebo to 8.1%. In a phase 3 trial comparing baricitinib and RDV combo treatment versus placebo plus RDV, hospitalized COVID19 patients treated with the combo drugs recovered faster than those treated with RDV alone (7 days vs. 8 days). The proportion of patients who died by Day 29 was 4.7% for the combo drug group compared to 7.1% for the RDV alone [35]. Another phase 3 clinical trial showed similar survival and improvements of the hospitalized COVID19 patients treated with either baricitinib plus RDV or dexamethasone plus RDV, but dexamethasone was associated with significantly more adverse events [36].

The immune modulatory drugs are only indicated for severe COVID19 cases that require hospitalization, and logically, they are best used in combination with anti-viral therapies. As for treating the vast majority of SARS-CoV-2 infections, as well as for preventing SARS-CoV-2 infections, HDA drugs will be the primary therapy in the future.

SARS-CoV-2 infection requires multiple host factors, and the best-characterized ones include: the ACE2 receptor, the heperan sulfate proteoglycan (HSPG) co-receptors that form electrostatic attachments with the virus on the airway surface and exponentially enhance the chance for the virus-ACE2 interaction [37], and the human type II transmembrane serine proteases (TTSPs), including TMPRSS2, TMPRSS11D, and TMPRSS13, that cleave the viral spike protein to activate membrane fusion and endocytosis [38]. Some of the HDTx and HDA approaches under development now target these host factors.

Inhibitors of the human serine proteases, such as aprotinin and Camostat, are effective against respiratory viruses, including SARS-CoV-2 and influenza [39, 40], and have long been safely used to treat inflammatory conditions such as acute pancreatitis, sepsis, and ARDS. Both drugs are being tested in clinical trials for COVID19 treatment. In a recent phase 3 clinical trial, inhaled aprotinin successfully treated hospitalized COVID19 patients by reducing oxygen requirements and shortening hospital stay by 5 days (p = 0.003) [41].

A new TTSP inhibitor, N-0385, have also demonstrated that a HDA candidate can be a potential pan-SARS-CoV-2 prophylactic and therapeutic. N-0385 is a chemical compound that inhibits human TMPRSS2, matriptase, and TMPRSS1. It inhibits various SARS-CoV-2 variants in vitro. In the K18-hACE2 model, intranasal treatment by N-0385 effectively treated severe COVID19 and prevented death, and efficacy was observed even after a single post-infectious treatment [42].

To fortify the resistance barrier against SARS-CoV-2 variants, HDA and HDTx therapies can be synergistically combined as the former can quickly control the viral load and the later may mitigate the pathogenic host responses to the virus. Additionally, HDA and HDTx approaches can be further strengthened through a multi-functional drug design which may enable a drug to simultaneously inhibit multiple essential steps in a viral life cycle as well as to offer variant agnostic treatment. As an example, a multifunctional drug candidate named *ResCovidin*™, which is under development by Abimmune Biopharma, has been designed to simultaneously mask all the key ports of entry recognized by the SARS-CoV-2 virus—ACE2, HSPG, & TTSPs (unpublished results). Although SARS-CoV-2 may mutate to overcome a certain step, to subvert them all, the virus would have to change into a fundamentally different virus.

To ensure safety of novel HDTx and HDA candidates, the host targets, the drug functional moieties, and the drug delivery methods all need to be carefully chosen and designed. For example, we have deemed ACE2 a safe target, at least when it is located on the airway surface, because this carboxypeptidase has minimal signaling function in the respiratory epithelium and its catalytic pocket is separated from the SARS-CoV-2 binding site, thus one can block the SARS-CoV-2 binding site without impairing ACE2’s normal enzymatic function [43]. Besides specifically targeting the viral binding site on ACE2, the receptor blocking moiety of *ResCovidin*™ is further designed for both enhanced affinity as well as diminished risk of off-target effects (data not shown). Finally, *ResCovidin*™ will be delivered through nasal spray or inhalation so that the drug will only function topically on the airway surface, thereby ensuring the best safety and efficacy profile.

The COVID19 pandemic heralds a new era of “superbugs”, a new paradigm is thus needed to combat them. The success of the mRNA vaccines has strengthened our believe that addressing the biggest public health challenges of our time through bold and innovative scientific endeavors can lead to immeasurable benefits to the world.

## Data Availability

Not applicable.
